# Redescription and affinities of *Hulsanpes perlei* (Dinosauria, Theropoda) from the Upper Cretaceous of Mongolia

**DOI:** 10.7717/peerj.4868

**Published:** 2018-05-28

**Authors:** Andrea Cau, Daniel Madzia

**Affiliations:** 1 Geological and Palaeontological Museum “G. Capellini”, Bologna, Italy; 2 Institute of Paleobiology, Polish Academy of Sciences, Warsaw, Poland

**Keywords:** Cretaceous, Halszkaraptorinae, Dromaeosauridae, *Hulsanpes perlei*, Mongolia, Theropoda

## Abstract

*Hulsanpes perlei* is an enigmatic theropod dinosaur from the Baruungoyot Formation (?mid- to upper Campanian, Upper Cretaceous) of Mongolia. It was discovered in 1970, during the third Polish-Mongolian paleontological expedition to the Nemegt Basin. The taxon is known based on a partial braincase and an incomplete right hindlimb. However, the braincase fragment has never been described nor illustrated. We redescribe all elements that form the holotype of *Hulsanpes* and discuss the affinities of this taxon. The braincase fragment is interpreted as belonging to the inner ear region, and includes the floccular recess and part of the labyrinth. *Hulsanpes perlei* is confirmed as a valid taxon, diagnosed by a unique combination of metatarsal characters, including two autapomorphies. Historically, it represents the oldest record of the recently-established clade Halszkaraptorinae. Our findings identify subcursorial adaptations for *Hulsanpes*, shared with *Mahakala,* and differentiating them from *Halszkaraptor*. As such, appendicular disparity in the potentially sympatric halszkaraptorines suggest a reduced ecological overlap among these taxa, which may explain the co-occurrence of multiple species of this clade during the latest Cretaceous in what is now the Nemegt Basin.

## Introduction

The Polish-Mongolian paleontological expeditions to the Gobi Desert, conducted between 1963 and 1971, have yielded rich and diverse assemblages of Late Cretaceous vertebrates, including numerous previously unrecognized dinosaurs (Osmólska & Roniewicz, 1970; Nowiński, 1971; Maryańska & Osmólska, 1974; Maryańska & Osmólska, 1975; Borsuk-Białynicka, 1977; Maryańska, 1977; Maryańska & Osmólska, 1981; Osmólska, 1982; Osmólska, 1987). Subsequent studies have revealed that some of these specimens are particularly enigmatic. These included, for example, the gigantic ornithomimosaur *Deinocheirus mirificus* (Osmólska & Roniewicz, 1970), the titanosaurs *Nemegtosaurus mongoliensis* (Nowiński, 1971) and *Opisthocoelicaudia skarzynskii* (Borsuk-Białynicka, 1977), and the tiny theropod *Hulsanpes perlei* Osmólska (1982). While the enigmas surrounding *Deinocheirus* and the potential synonymy of *Nemegtosaurus* and *Opisthocoelicaudia* are being gradually solved (Lee et al., 2014; Madzia & Borsuk-Białynicka, 2014; Currie et al., 2018), our perception of *Hulsanpes* is still limited by incomplete knowledge of the true identity of its type material.

Osmólska (1982) based the original description of *Hulsanpes* mainly on a partially complete right metatarsus, though she also mentioned an “?otico-occipital fragment of skull” as a part of the holotype. The braincase fragment, however, has never been described nor illustrated.

The only known specimen of *Hulsanpes* was discovered during the third Polish-Mongolian expedition in 1970, at the Khulsan locality (Nemegt Basin, Mongolia), in the strata forming the Campanian Baruungoyot Formation.

Based on a roughly textured external surface of the metatarsus, its relatively small size, and overall slenderness, Osmólska (1982) interpreted the type specimen as an immature individual. Focusing on the morphology of the metatarsus and the preserved second toe phalanges, she classified *Hulsanpes* as a deinonychosaurian, and tentatively referred it to Dromaeosauridae. Since its original description, *Hulsanpes* has received only limited attention, mainly due to its extremely fragmentary nature. Gauthier (1986) included *Hulsanpes* in the newly-established Maniraptora. Although Ostrom (1990) briefly listed *Hulsanpes* among dromaeosaurids in his revision of the clade, Norell & Makovicky (2004) found no dromaeosaurid synapomorphies in *Hulsanpes*. The phylogenetic analysis of Senter et al. (2004) supported deinonychosaurian affinities for *Hulsanpes*, inferring its closer relationships to dromaeosaurids than to birds. Cau et al. (2017) provided an emended differential diagnosis of *Hulsanpes* and inferred it as the sister taxon of *Mahakala* (Turner et al., 2007; Turner, Pol & Norell, 2011), within a newly recognized lineage of peculiar dromaeosaurids that they named Halszkaraptorinae.

Here, we provide a redescription of *Hulsanpes perlei*, supplementing the original description of Osmólska (1982). Specifically, we provide the first assessment and illustrations of the braincase fragment, and discuss the affinities of this taxon.

## Material and Methods

### Material

The study is based on personal examination of the type specimen of the paravian theropod *Hulsanpes perlei* consisting of a partial braincase and an incomplete right hindlimb. The material is housed at the Institute of Paleobiology of the Polish Academy of Sciences under the catalog number ZPAL MgD-I/173.

### Photographs

The material was photographed using digital single-lens reflex camera Nikon D1X. Additional close-up pictures were taken through the binocular microscope Nikon SMZ800 using Nikon 1 J3.

### Anatomical terminology

We follow the terminology used in Witmer (1990), Barsbold & Osmólska (1999), and Balanoff, Bever & Norell (2014).

### Phylogenetic analysis

The phylogenetic affinities of *Hulsanpes* were investigated using the data set of Cau et al. (2017).

The data set was analyzed using TNT 1.5 (Goloboff, Farris & Nixon, 2008). First, we performed a parsimony analysis using the “New Technology” search, with 100 replications and default parameters. Then, we explored the inferred shortest tree islands through the “Traditional Search” analyses, again using default parameters, and saving all shortest trees reconstructed. Nodal support was calculated in TNT, saving all trees up to 10 steps longer than the most parsimonious results.

Phylogenetic nomenclature of coelurosaurs follows Cau et al. (2017).

## Systematic Paleontology

**Table:** 

Theropoda Marsh, 1881
Maniraptora Gauthier, 1986
Dromaeosauridae Matthew & Brown, 1922
Halszkaraptorinae Cau et al., 2017
*Hulsanpes perlei* Osmólska, 1982

### Holotype

**Figure 1:**
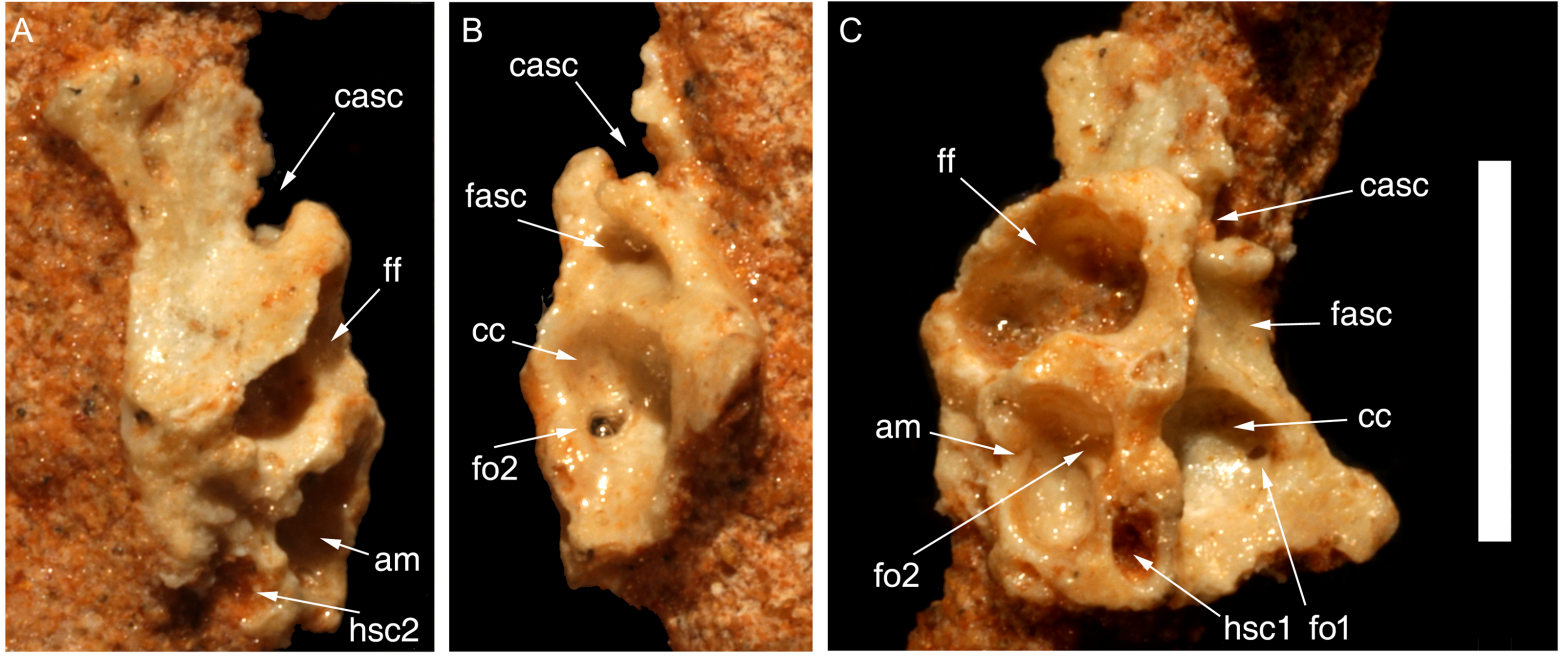
Partial right medial wall of braincase of *Hulsanpes perlei* (ZPAL MgD-I/173) in anterior view (A), posterior view (B), and medial view (C). Abbreviations: am, ampulla; casc, cleft of the anterior semicircular canal; cc, common crus; fasc, fossa of the anterior semicircular canal; ff, floccular fossa; fo, foramen; hsc, passage of the horizontal semicircular canal. Scale bar: 5 mm. Photograph credits: Daniel Madzia.

**Figure 2:**
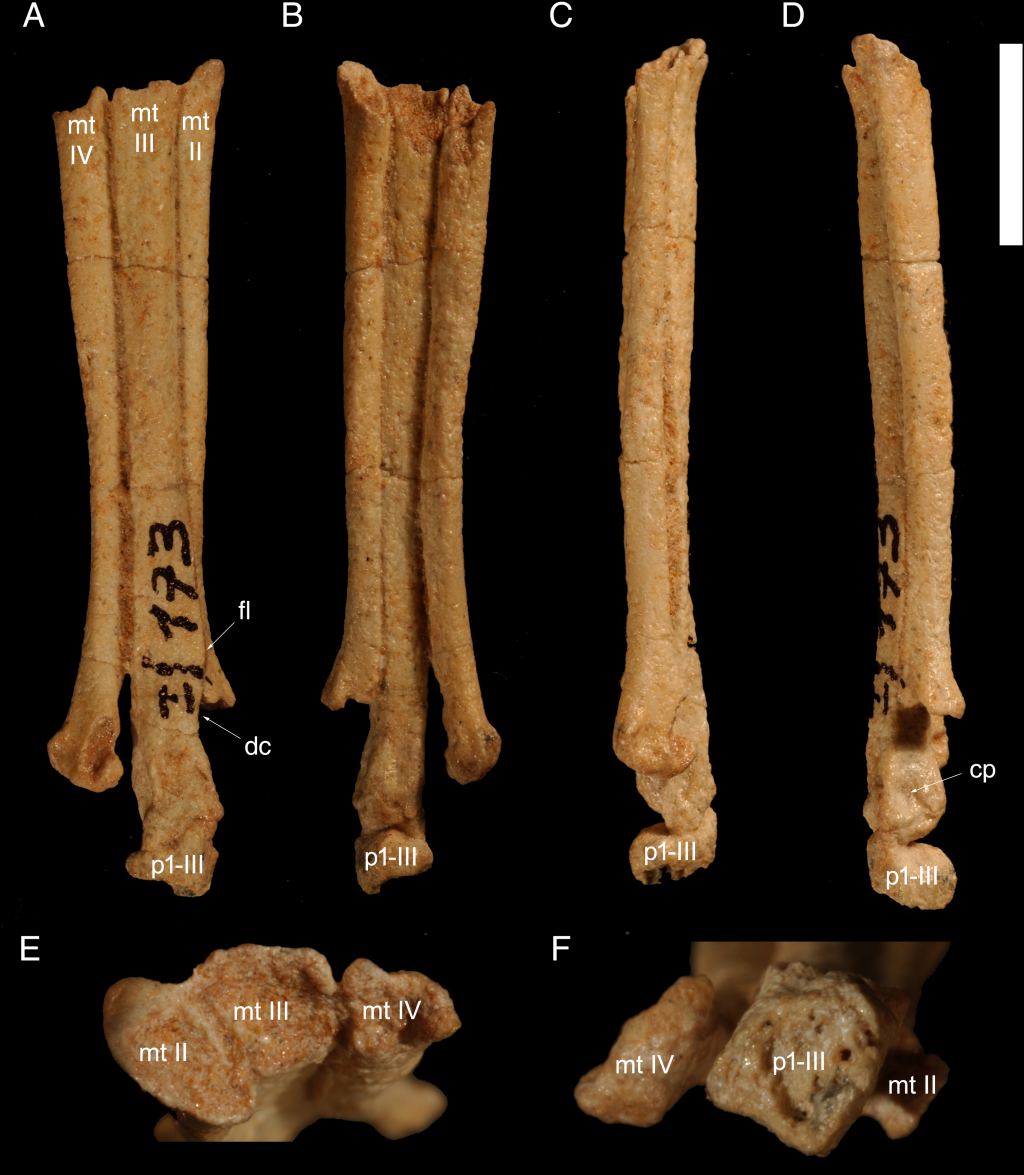
Right metatarsals II–IV and pedal phalanx p1-III of *Hulsanpes perlei* (ZPAL MgD-I/173). Extensor view (A), flexor view (B), lateral view (C), medial view (D), proximal view (E), distal view (F) . Abbreviations: cp, collateral pit; dc, shaft constriction distal to flange; fl, medial flange of metatarsal III; mtII-IV, metatarsals II–IV; p1-III, pedal phalanx p1-III. Scale bar in A–D: 10 mm. Photograph credits: Daniel Madzia.

**Figure 3:**
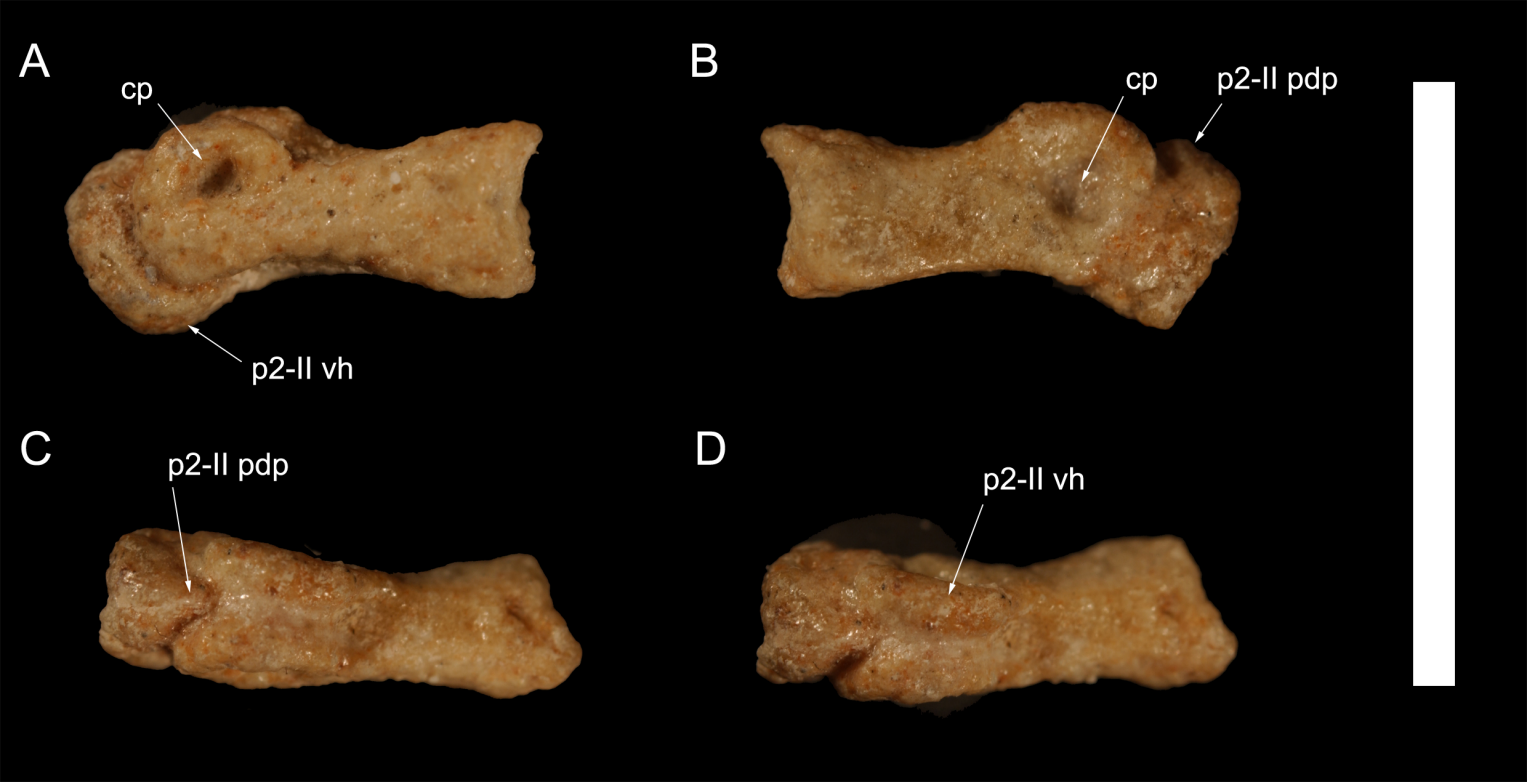
Right pedal phalanx P1-II and proximal end of pedal phalanx p2-II of *Hulsanpes perlei* (ZPAL MgD-I/173). Medial view (A), lateral view (B), dorsal view (C), ventral view (D). Abbreviations: cp, collateral pit; p2-II pdp, posterodorsal process of phalanx p2-II; p2-II vh, ventral heel of phalanx p2-II. Scale bar: 10 mm. Photograph credits: Daniel Madzia.

ZPAL MgD-I/173 (Figs. 1–3, Table 1): right metatarsals II–III–IV, and partial right pedal phalanx p1-III, all in articulation (Fig. 2). The material also includes a right pedal phalanx p1-II attached to the partial proximal end of phalanx p2-II (Fig. 3), and an “?otico-occipital fragment of skull (probably pertaining to the specimen)” (Osmólska, 1982: 441). The latter element (Fig. 1), an incomplete right medial wall of the braincase, is referable to the same size class as the appendicular elements, and is cataloged under the same number as the metatarsus. Following Osmólska (1982), all elements are referred to the same individual.

**Table 1:** Selected measurements (in mm) of known halszkaraptorines.

Taxon/specimen number	Frontal	Femur	Tibia	Metatarsal II	Metatarsal III	Metatarsal IV	Pedal phalanx p1-II
*Hulsanpes perlei* ZPAL MgD-I/173	–	–	–	34 (79%)	39^*^ (77%)	36^*^ (77%)	6.5 (76%)
*Halszkaraptor escuilliei* MPC-D 102/109	24.8	76.2	105.5	43	50.2	46.7	8.6
*Mahakala omnogovae* MPC-D 100/1033	25.2 (102%)	79 (104%)	110 (104%)	–	52 (104%)	–	–

**Notes.**

* indicates estimated value.

In *Hulsanpes* and *Mahakala*, % indicates ratio relative to homologous element in *Halszkaraptor escuilliei*.

### Locality and age

Khulsan locality, Nemegt Basin, Gobi Desert, Mongolia; Baruungoyot Formation; Upper Cretaceous, ?mid- to upper Campanian, (Jerzykiewicz, 2000; Fanti, Cantelli & Angelicola, 2018; Eberth, 2018).

### Emended diagnosis

A paravian theropod with the following unique combination of features (autapomorphies among Dromaeosauridae and Halszkaraptorinae marked with *): distal third of metatarsal II shaft extensively overlapped by metatarsal III in extensor view*; proximal shaft of metatarsal III unconstricted and wider than adjacent metatarsals*; metatarsal III–IV contact straight in extensor view; distal end of metatarsal IV diverges laterodistally.

### Remarks

Osmólska (1982) diagnosed *Hulsanpes* as a “theropod with [1] functionally tridactyl pes; [2] metatarsus slender (width: length ratio 0.16); [3] metatarsal III not wedged in anterior view; [4] metatarsals II–IV subequally thick; [5] second pedal digit specialized”. This diagnosis is not adequate for differentiating *Hulsanpes* from other theropods, and specifically, from other paravians. Features [1], [3], and [4] are theropod symplesiomorphies (Holtz, 1998). Feature [2] is an ontogeny- and size-related ratio, with limited taxonomic significance (e.g., Holtz, 1995; Currie, 2003). Feature [5] alludes to the features in the second pedal digit allowing hyperextention (Ostrom, 1969), a complex of characters that is homoplastic among paravians (see Turner, Makovicky & Norell, 2012; Cau, Brougham & Naish, 2015). Cau et al. (2017) provided an emended differential diagnosis of *Hulsanpes* that distinguishes the type metatarsus from that of *Halszkaraptor* and *Mahakala*. The diagnosis provided here covers the complete set of features present in the type specimen that differentiate *Hulsanpes* from all theropods known from metatarsal elements.

## Description

### Braincase fragment

**Figure 4:**
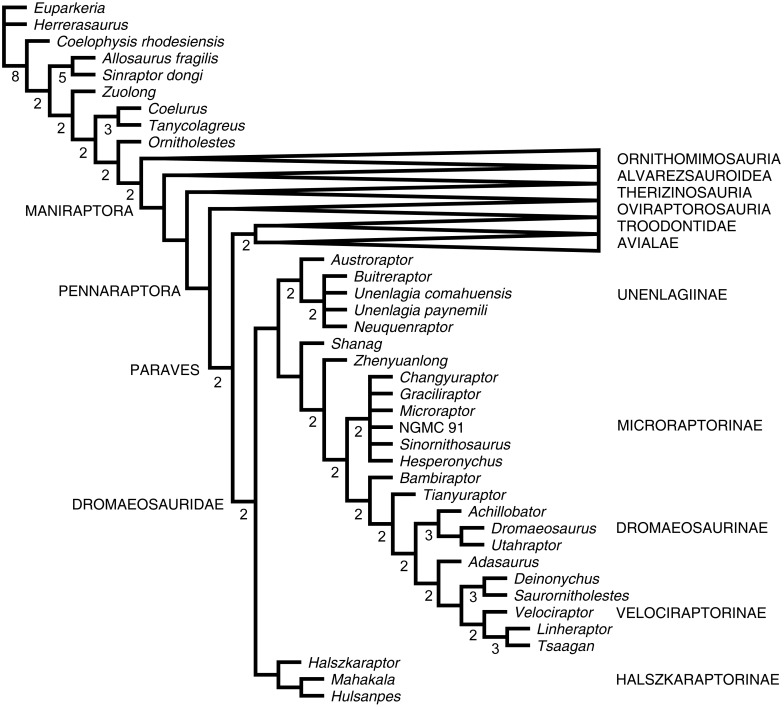
Affinities of *Hulsanpes perlei*. Strict consensus of the shortest trees reconstructed in the phylogenetic analysis. Larger clades (in capital letters) collapsed for brevity. Numbers adjacent to nodes indicate Decay Index values >1.

Osmólska (1982) reported an “?otico-occipital fragment of skull” among the holotype material of *Hulsanpes perlei*, but did not provide its description nor illustration. The element is a tiny (10 mm long) fragment of bone, partially embedded in the sandstone matrix (Fig. 1). The braincase fragment is irregular in shape, and all sutural contacts with adjacent elements are lost. It shows a complex pattern of foramina and blind fossae. Based on comparisons with the braincases of other maniraptorans (e.g., *Archaeopteryx*, Rauhut, 2014; *Conchoraptor*, Balanoff, Bever & Norell, 2014; *Erlikosaurus*, Lautenschlager et al., 2012; *Incisivosaurus*, Balanoff et al., 2009; *Mahakala*, Turner, Pol & Norell, 2011; *Sinovenator*, Xu et al., 2002; late-diverging troodontids, Currie & Zhao, 1994; *Velociraptor*, Barsbold & Osmólska, 1999; Norell, Makovicky & Clarke, 2004), we interpret the exposed surface of this braincase fragment as the medial surface of the right prootic-opisthotic. In particular, the largest fossa in the braincase fragment of ZPAL MgD-I/173 appears too deep and sharply-bordered for being interpreted as a tympanic recess (Witmer, 1990; O Rauhut, pers. com., 2018), and is more likely interpreted as the floccular fossa (e.g., Norell, Makovicky & Clarke, 2004; Lautenschlager et al., 2012). Following this interpretation, the longest axis of the exposed surface of the fragment is oriented vertically (Fig. 1). The anteromedial part of the exposed surface is partially eroded, and the extent of the missing parts is uncertain. The posterior and lateral margins of the exposed bone are better preserved. Starting from the dorsal margin and moving clockwise, the main elements of the exposed surface are: (1) a large and deep fossa oriented anteromedially, bordered ventrally and posteriorly by a thick ridge (“ff” in Fig. 1); (2) a dorsomedially-oriented cleft, which may represent the remnant of a foramen (“casc” in Fig. 1); (3) a large elliptical fossa (“cc” in Fig. 1) that houses a foramen (“fo1” in Fig. 1); (4) an “8”-shaped fossa (“am” in Fig. 1) housing a foramen (“fo2” in Fig. 1); and (5) a large elliptical foramen facing medially and placed between the fossae (3) and (4) (“hsc1” in Fig. 1).

The wide anterodorsal opening is interpreted as the fossa for the flocculus cerebelli. In *Mahakala*, *Tsaagan* and other dromaeosaurids, the floccular fossa is relatively large, deep, and oriented anteromedially (Currie, 1995; Norell, Makovicky & Clarke, 2004; Turner, Pol & Norell, 2011). In ZPAL MgD-I/173, this fossa is elliptical in medial view, with the long axis oriented anterolaterally. It is separated posteroventrally from the other fossae by a thickened margin. The anterodorsal roof of the floccular fossa is thinner and gently concave in anterodorsal view. Posterodorsally, the medial margin of the roof of the floccular fossa is raised dorsomedially, and partially overlaps a cleft. The floor of the floccular fossa is covered by sediment.

In the posterodorsal corner of the preserved fragment, a rounded cleft connects the dorsal surface of the anterodorsal roof of the floccular fossa with the posterior margin of the fragment. It is likely that originally the cleft was a closed foramen. The posterior surface of the braincase fragment is excavated by two fossae aligned dorsoventrally. The dorsal fossa is smaller and shallower than the ventral fossa. It communicates with the posterodorsal cleft through an incision. The ventral fossa is oriented posteromedially. It is suboval in medial view and houses two foramina. The first foramen is placed in the posterior border of the fossa and is directed laterally (“fo1” in Fig. 1). The second foramen penetrates the vertical bony wall placed perpendicularly to the floccular fossa, and connects the posteroventral fossa with the anteroventral fossa (“fo2” in Fig. 1).

A large foramen, roughly elliptical in medial view, opens in the ventral end of the bony strut separating the anteroventral and posteroventral fossae (“hsc1” in Fig. 1C). Another foramen, less completely preserved but roughly similar in both size and dimension to the latter, is placed in the anteroventral corner of the preserved fragment (“hsc2” in Fig. 1A).

The anterior and posterior crurae of the anterior semicircular canal delimit the medial margins of the floccular recess in other maniraptorans (e.g., *Conchoraptor*, Balanoff, Bever & Norell, 2014; *Tsaagan*, Norell et al., 2006; therizinosaurids, Lautenschlager et al., 2012). We thus interpret the shallow concavity on the anterodorsal surface of the bone fragment, the posterodorsal cleft and the sulcus along the posterodorsal fossa as the impressions of the anterior semicircular canal surrounding the floccular recess. Following this interpretation, the two large ventral fossae placed ventrally to the floccular fossa (“am” and “cc” in Fig. 1) may represent the impressions of the lateral part of the ampulla and the common crus of the inner ear labyrinth (e.g., (Balanoff, Bever & Norell, 2014), Fig. 3). We tentatively interpret the two large elliptical foramina placed symmetrically to the anteroventral fossa (“hsc1” and “hsc2” in Fig. 1) as the passages of the horizontal semicircular canal.

### Metatarsus

The second, third, and fourth metatarsals of the right pes are almost completely preserved and in articulation. The proximal end of all three metatarsals is largely eroded, and only the anteromedial corner of metatarsal II is still preserved. The latter allows for accurate measurements of metatarsal II, and provides a proximal landmark for accurate estimation of the length of the other two metatarsals. The metatarsus is gracile, about 6.25 times longer than wide at its narrowest point. The metatarsus as a whole appears constricted at about 2/3 to 3/4 of its length: this is mostly due to the conspicuous overlap of metatarsal III on the distal half of metatarsal II, which reduces the participation of the latter on the extensor surface of the metatarsus, and the lateral divergence and partial torsion of metatarsal IV distal shaft. The eroded proximal surfaces of the metatarsals show the thin cortex and the large medullary cavities, widespread among theropod long bones. Both, the metatarsals II and IV are appressed to metatarsal III along most of their proximodistal lengths, and diverge from the middle metatarsal in the final fifth of its length. As outlined by Osmólska (1982), the external surface of the long bones is roughly textured, which is an ontogeny-related feature widespread among immature theropod specimens (e.g., *Scipionyx samniticus*, holotype, Dal Sasso & Maganuco, 2011; *Eosinopteryx brevipenna*, holotype, A Cau, pers. obs., 2015).

Metatarsal II is elliptical in proximal view, with the long axis oriented posterolaterally-anteromedially. The shaft is straight and subparallel to that of the third in flexor (‘ventral’) view. In extensor (‘dorsal’) view, the distal third of the bone is widely overlapped by a medial distal flange of metatarsal III. Although a distal medial flange of metatarsal III, that partially overlaps metatarsal II, is present in other paravians (e.g., *Mahakala*, Turner, Pol & Norell, 2011; *Sinovenator*, Xu et al., 2002; *Neuquenraptor*, Brissón Egli et al., 2017), the extensive overlap present in *Hulsanpes* is absent in other dromaeosaurids, and recalls the condition observable in many late-diverging troodontids (e.g., *Talos*, Zanno et al., 2011, *Philovenator*, Xu et al., 2012). Nevertheless, in those troodontids, the distal half of metatarsal II is markedly constricted transversally relative to the proximal half (e.g., Xu et al., 2012), whereas in *Hulsanpes*, the width of metatarsal II is uniform along the shaft. Since both metatarsals II and III do not show any sign of deformation along their mutual contact, the relative placement of metatarsal II relative to metatarsal III is not a preservational artifact, and represents an autapomorphy of *Hulsanpes perlei*. As in other halszkaraptorines (Cau et al., 2017) and in avisaurids (Chiappe, 1993), the extensor surface of the shaft is moderately convex transversally, but lacks the prominent ridge-like margin present in some avialans (e.g., *Balaur*, *Mystiornis*, *Vorona*, see Cau, Brougham & Naish, 2015). The flexor surface of the shaft of metatarsal II is uniformly convex along the mediolateral axis, and does not show the distinct ridge or flange extending along the medial margin of the flexor surface that is present in some avialans (e.g., *Hollanda*, Bell et al., 2010; *Mystiornis*, Kurochkin et al., 2010). The distal end of the shaft is missing. The distalmost portion of the preserved part of the shaft is bent medially from the longitudinal axis of the metatarsus: this suggests that the distal end of the bone was exposed in extensor view and placed symmetrically to that of metatarsal IV.

Metatarsal III is the longest and most robust element of the foot. The preserved proximal end of the shaft is quadrangular and comparable in size to the other two metatarsals. This condition differs from that in many coelurosaurs, where the proximal end of the metatarsal III is reduced in size compared to the adjacent metatarsals (Holtz, 1995). As in *Mahakala* (Turner, Pol & Norell, 2011) and *Halszkaraptor* (Cau et al., 2017), metatarsal III of *Hulsanpes* is not constricted proximally in both extensor and flexor views. In many Late Cretaceous coelurosaurs from Central Asia, including troodontids (Xu et al., 2002; Xu et al., 2012; Zanno et al., 2011), most oviraptorosaurs (e.g., *Elmisaurus*, Osmólska, 1981), parvicursorines (Perle et al., 1994), ornithomimids (Osmólska, Roniewicz & Barsbold, 1972), and tyrannosaurids (Mader & Bradley, 1989), metatarsal III shows a variable degree of transversal constriction on the proximal end of shaft (Holtz, 1995). Among Mongolian coelurosaurs, metatarsal III is proximally unconstricted in eudromaeosaurs (Norell & Makovicky, 1999), avialans (e.g., *Hollanda*, Bell et al., 2010), deinocheirids (Lee et al., 2014), therizinosaurids (Perle, 1979) and some oviraptorids (e.g., *Khaan*, Balanoff & Norell, 2012). In extensor view, metatarsal III is straight for most of its length, with subparallel medial and lateral margins. The distal third of the medial margin is sigmoid in extensor view, due to the development of a medial flange (‘tongue-like process’ of Brissón Egli et al., 2017) that overlaps metatarsal II. This flange is separated from the distal end by a constriction. This combination of features is shared with *Mahakala* (Turner, Pol & Norell, 2011), *Neuquenraptor* (Brissón Egli et al., 2017), and *Sinovenator* (Xu et al., 2002). The proximal half of the extensor surface of metatarsal III is transversally convex, as in *Mahakala* and *Halszkaraptor* (Cau et al., 2017). Among paravians, this condition differs from unenlagiines and troodontids, where the extensor surface of metatarsal III is plantarly displaced in its proximal half relative to the adjacent metatarsals (Zanno et al., 2011; Brissón Egli et al., 2017). The trochlea of metatarsal III is dorsoventrally (along the extensor-plantar direction) low and proximodistally elongate, similar to *Halszkaraptor* and *Mahakala* in overall proportions (e.g., Turner, Pol & Norell, 2011, fig. 33). Its distal end is slightly bent medially relative to the rest of the bone. The extensor surface of its distal end bears a shallow fossa that is confluent with the extensor part of the intercondylar groove. The distal articular facet is mostly overlapped by the phalanx III-1, still in articulation, and is ginglymoid as in *Mahakala* and most dromaeosaurids (Norell & Makovicky, 1999). The lateral condyle is larger and extended more distally than the medial condyle. The collateral pits are wide and shallow.

Metatarsal IV is well-preserved; only the proximal end and the medial margin of the distal articulation are missing. The bone is shorter than the third metatarsal and is gently bent laterally in its distal half. Its shaft extends along the lateroventral margin of the metatarsal III, and is not significantly overlapped by the latter as is metatarsal II. The ventral surface of the metatarsal is broadly rounded transversally. As in *Mahakala*, it does not bear the distinct ventrolateral flange present in other Mongolian dromaeosaurids (e.g., Norell & Makovicky, 1999). Its distal end is rotated counter-clockwise relative to the mediolateral axis of its proximal portion so that the extensor surface of its distal articulation is oriented anterolaterally relative to the same surface of the metatarsal III. The distal end is dorsoventrally compressed (i.e., it is twice as wide as deep in distal view), similar to the condition in eudromaeosaurs and many avialans (Cau, Brougham & Naish, 2015). The distal end appears subtriangular in distal view (Fig. 3F), mostly due to erosion of the medial condyle. The distal articular surface is not ginglymoid and a shallow extensor fossa widely separates the two poorly-differentiated condyles.

### Pedal phalanges

Three pedal phalanges of the right pes are preserved (P1-II, P2-II and P1-III), but only P1-II is complete. P1-II is moderately elongate, being three times as long as the trochlear eminence. Its proximal articular surface is moderately concave in lateral and medial views, and is overlapped by a lip-like proximodorsal projection. The trochlea is ginglymoid and equally projected anteriorly and dorsally (i.e., it describes a ∼270°arc along the extensor and distal directions). The collateral ligament pits are asymmetrically developed, with the medial pit deeper and more dorsally placed than the lateral pit.

Only the proximal end of the phalanx P2-II is preserved. The bone is tightly adherent to the distal end of phalanx P1-II, in natural articulation. The phalanx bears a distinct proximodorsal lip that is less prominent than the serially homologous element in the preceding phalanx. The ventral margin of the proximal facet is extended posteriorly as an elongate and narrow heel (about twice longer than wide) placed symmetrically relative to the ventral surface of the first phalanx. The proximoventral heel in this phalanx is thus comparable to the condition in *Mahakala* and eudromaeosaurs than to the smaller and asymmetrical lip present in unenlagiines, microraptorines, and troodontids (Makovicky, Apesteguía & Agnolín, 2005; Turner, Pol & Norell, 2011; Brissón Egli et al., 2017).

P1-III is badly eroded and only its proximal end, still in articulation with the third metatarsal, is preserved.

## Results

The phylogenetic analysis of the data from Cau et al. (2017), updated for *Hulsanpes*, reconstructed >99.999 shortest trees of 6458 steps each (CI: 0.2306, RI: 0.6054). The strict consensus of the shortest trees inferred is broadly consistent with the result in Cau et al. (2017), as it confirms the pectinate series of avialan sister groups reconstructed in the previous iteration of this data set (Fig. 4). *Hulsanpes* is inferred as the sister taxon of *Mahakala*, within a clade of dromaeosaurids formed by the two taxa and *Halszkaraptor*. Such topology repeats the results obtained by Cau et al. (2017) and supports the establishment of Halszkaraptorinae.

Dromaeosauridae is diagnosed by the following series of unambiguous synapomorphies: anterior tympanic recess placed anteriorly on the basipterygoid process (unknown in *Hulsanpes*); presence of a ventral flange on paroccipital process (unknown in *Hulsanpes*); absence of basal constriction between crown and root in teeth (reversal in *Microraptor*, unknown in *Hulsanpes*); dorsal and ventral margin of dentary subparallel for most of their length (unknown in *Hulsanpes*); cervical centra not extending posteriorly beyond the neural arch (unknown in *Hulsanpes*); six sacral vertebrae (unknown in *Hulsanpes*); short radius, no longer than 3/5 of the humerus length (unknown in *Hulsanpes*); ischium 5 times longer than its minimum anteroposterior diameter (unknown in *Hulsanpes*); short pedal phalanx P1-II, no longer than three times the length of its trochlea (present in *Hulsanpes*); shortened pedal phalanx p2-II, no longer than twice the length of its trochlea (unknown in *Hulsanpes*).

The clade Halszkaraptorinae is diagnosed by the fusion of the cervical ribs to centra (unknown in *Hulsanpes*); presence of a distinct prezygocostal lamina in proximal caudal vertebrae (unknown in *Hulsanpes*); horizontal orientation of the zygapophyseal facets in the proximal caudal vertebrae (unknown in *Hulsanpes*); abrupt reduction in size of the proximalmost caudal neural spines (unknown in *Hulsanpes*); presence of a prominent supratrochanteric process on ilium that is curved dorsolaterally (unknown in *Hulsanpes*); presence of a marked lateral ridge along the posterior margin of the distal end of the femur (unknown in *Hulsanpes*); unconstricted proximal end in metatarsal III (present in *Hulsanpes*); presence of a transversely convex dorsal surface of metatarsal III (present in *Hulsanpes*).

The sister-taxon relationship between *Hulsanpes* and *Mahakala* is supported by one unambiguous synapomorphy: presence of a lateral flange on metatarsal III overlapping metatarsal II.

## Discussion

### Validity and affinities of the taxon

Although it is based on a very fragmentary specimen, *Hulsanpes perlei* can be distinguished from all other theropods by the unique combination of features that characterizes its metatarsus. In particular, the relative robustness of the proximal shaft of metatarsal III, which is wider than both metatarsals II and IV, is an unusual feature among Late Cretaceous coelurosaurs and is shared exclusively with some enantiornithine avialans (Chiappe, 1993). It is noteworthy that the metatarsus of *Hulsanpes* shares other features with some Late Cretaceous enantiornithines, including a markedly convex extensor surface of the proximal end of metatarsal III (although it is also shared with other halszkaraptorines, Cau et al., 2017), a planar arrangement of metatarsals II–IV in proximal view, with metatarsal III not displaced plantarly, a marked compression of the shaft of metatarsal IV, and a laterally-bowed distal end of metatarsal IV shaft (Chiappe, 1993). Nevertheless, the result of the phylogenetic analysis does not support an avialan (or enantiornithine) affinity for *Hulsanpes.* Enforcing an avialan placement for *Hulsanpes* (i.e., as closer to *Meleagris* than troodontids and dromaeosaurids), the shortest trees inferred are four steps longer than the most parsimonious topologies (Fig. 4). We thus provisionally reject an avialan status for *Hulsanpes* and refer it to Dromaeosauridae. In other dromaeosaurids, the distal half of metatarsal II is only partially (if not at all) overlapped by metatarsal III. *Hulsanpes perlei* is therefore considered a valid taxon.

### Ontogenetic stage

The metatarsus and toe phalanges of *Hu. perlei* are about 75–80% the size of the same elements of the type of *Halszkaraptor escuilliei*, a nearly complete subadult individual inferred to be at least one year old at the time of its death (Cau et al., 2017). Thus, considering the size of ZPAL MgD-I/173 and the roughly textured surface of its long bones, which suggests an early stage of post-hatchling development (Dal Sasso & Maganuco, 2011), we concur with Osmólska (1982) that ZPAL MgD-I/173 is an immature individual.

### Ecology

The metatarsus is the only available skeletal element known in all three halszkaraptorine taxa. Based on measurements in Turner et al. (2007, supplementary information) and Turner, Pol & Norell (2011), Cau et al. (2017) reported that the metatarsus of *Mahakala* is relatively more elongate than in the comparably-sized *Halszkaraptor* (i.e., metatarsal III of *Mahakala* being 104% longer than the femur, while in *Halszkaraptor* the length of the same element is about 80% the length of the femur). Although such value would indicate an apomorphically elongate metatarsus in *Mahakala* compared to other paravians (Holtz, 1995; Dececchi & Larsson, 2013), Turner et al. (2007) and Turner, Pol & Norell (2011) did not mention this feature among the diagnostic features of that taxon. Nevertheless, the length of the metatarsus of *Mahakala* reported in Turner et al. (2007; 2011: 82 mm) differs significantly from the value reported for the same specimen in Dececchi & Larsson (2013: 52 mm). The latter value is comparable to the length of the metatarsus in *Ha. escuilliei* (i.e., 50.2 mm, Cau et al., 2017, supplementary information), is more consistent with the size of the metatarsus of *Mahakala* estimated from the published photographs (Turner et al., 2007, fig. 2E; Turner, Pol & Norell, 2011, figs. 33 and 35), and is confirmed by direct examination of a cast of the specimen (A. Dececchi, 2018, pers. com. to AC). We therefore consider the 82 mm value reported in Turner et al. (2007) and Turner, Pol & Norell (2011) as a typographical error, and follow the metatarsus length value reported in Dececchi & Larsson (2013). Accordingly, *Mahakala* and *Hulsanpes* share similar hindlimb proportions (i.e., tibia-femur ratio of 1.38-1.39; metatarsus-tibia ratio of 0.46–0.50; Table 1). Even assuming similar hindlimb proportions, the metatarsi of the three halszkaraptorines show morphological differences that may support distinct locomotory adaptations. Both *Hulsanpes* and *Mahakala* show a distinct flange on metatarsal III overlapping metatarsal II, a feature that is absent in *Halszkaraptor* (Cau et al., 2017). A similar feature is known in other coelurosaurs with a subarctometatarsalian foot (e.g., *Sinovenator*, *Neuquenraptor*; White, 2009), or with a fully arctometatarsalian condition (e.g., ornithomimids, tyrannosaurids, Osmólska, Roniewicz & Barsbold, 1972; Holtz, 1995; Brochu, 2003). This feature prevents relative torsion and dislocation of metatarsal II and III, and is related to an increased agility (Holtz, 1995; Snively, Russell & Powell, 2004). Using the strict consensus of the shortest trees inferred in our analysis as phyletic framework for character transition optimization, the subarctometatarsalian condition (i.e., the incipient constriction of the proximal end of metatarsal III; White, 2009) is inferred as a pennaraptoran synapomorphy. The last common ancestor of all halszkaraptorines lost this condition, convergently with eudromaeosaurs, and acquired a morphology similar to those of non-coelurosaurian theropods (i.e., the proximal end of metatarsal III being comparable in width to the adjacent metatarsals; Holtz, 1995). Among halszkaraptorines, the lineage leading to *Hulsanpes* and *Mahakala* acquired the flange on metatarsal III overlapping metatarsal II. Furthermore, the conspicuous overlap of metatarsal III on metatarsal II, seen exclusively in *Hulsanpes* among dromaeosaurids, is convergently acquired in arctometatarsalian troodontids (e.g., Zanno et al., 2011). Thus, unlike *Halszkaraptor*, both *Hulsanpes* and *Mahakala* show secondary acquisition of metatarsal features interpreted as cursorial adaptations (Holtz, 1995; Snively, Russell & Powell, 2004). The absence of cursorial adaptations in *Halszkaraptor* is consistent with the inference of the amphibious lifestyle in this taxon (Cau et al., 2017). Although limited to the metatarsus, the morphological diversity within Halszkaraptorinae supports some ecological diversification within this clade, between semi-aquatic and subcursorial forms.

## Conclusions

*Hulsanpes perlei* from the ?mid- to upper Campanian Baruungoyot Formation of Mongolia is a valid taxon of dromaeosaurid theropod, based on a fragment of the braincase and an incomplete right hindlimb, and diagnosed by a unique combination of features and two autapomorphies. The braincase fragment, however, has never been described nor illustrated. We redescribe all elements that form the holotype of *Hu. perlei* and discuss the affinities of this taxon, supporting its close relationships to *Mahakala* and *Halszkaraptor*. Discovered over 45 years ago, *Hulsanpes* represents the first record of the very recently established clade Halszkaraptorinae. Our findings suggest subcursorial adaptations for *Hu. perlei* shared with *Mahakala omnogovae*. Locomotory specializations in the potentially sympatric halszkaraptorines suggest a reduced ecological overlap (and, thus, limited source competition) among these taxa, which may explain the co-occurrence of multiple species of this clade during the latest Cretaceous in what is now the Nemegt Basin. The re-evaluation of this small and fragmentary theropod underlines the historical and scientific significance of the material collected during the Polish-Mongolian paleontological expeditions in the ’60s and ’70s of the 20th century.

##  Supplemental Information

10.7717/peerj.4868/supp-1 Supplemental Information 1 Phylogenetic data matrix Data matrix in .tnt format, with scores updated for Hulsanpes. Character list in Cau et al. (2017, supplementary data).

## Institutional abbreviations

MPC: Institute of Paleontology and Geology, Mongolian Academy of Sciences, Ulanbatar, Mongolia.
ZPAL: Institute of Paleobiology, Polish Academy of Sciences, Warsaw, Poland.

## Additional Information and Declarations

### Data Availability

The following information was supplied regarding data availability: The data matrix in TNT format has been included as a Supplementary File.
